# Microlocalization and clinical significance of stabilin-1^+^ macrophages in treatment-naïve patients with urothelial carcinoma of the bladder

**DOI:** 10.1007/s00345-019-02853-0

**Published:** 2019-07-13

**Authors:** Bo Wang, Hao Huang, Meihua Yang, Wenjuan Yang, Zhuowei Liu, Weibin Hou, Hong Zeng, Zhihua He, Tianxin Lin, Jian Huang

**Affiliations:** 1 Department of Urology, Sun Yat-sen Memorial Hospital, Sun Yat-sen (Zhongshan) University, Guangzhou, 510120 People’s Republic of China; 2 Guangdong Provincial Key Laboratory of Malignant Tumor Epigenetics and Gene Regulation, Sun Yat-Sen Memorial Hospital, Sun Yat-Sen University, Guangzhou, People’s Republic of China; 3 Department of Hematology, Sun Yat-sen Memorial Hospital, Sun Yat-sen (Zhongshan) University, Guangzhou, People’s Republic of China; 4 Department of Urology, Cancer Center, Sun Yat-sen (Zhongshan) University, Guangzhou, People’s Republic of China; 5 Department of Pathology, Sun Yat-sen Memorial Hospital, Sun Yat-sen (Zhongshan) University, Guangzhou, People’s Republic of China

**Keywords:** Urothelial carcinoma of the bladder, Tumor-associated macrophages, Stabilin-1, Microlocalization

## Abstract

**Purpose:**

Emerging evidence has shown that macrophages (Mφs) at different tumor sites have diverse clinical attributes. Stabilin-1 is a multi-functional scavenger marker for specialized tumor-associated Mφs. This study investigates the relationship between the density and microlocalization of stabilin-1^+^ Mφs within tumors and the clinical outcomes of patients with urothelial carcinoma of the bladder (UCB).

**Methods:**

In this retrospective study, 283 UCB patients who received radical cystectomy or transurethral resection were examined. Immunohistochemistry and immunofluorescence analyses were used to colocalize the expression of stabilin-1 with other markers for Mφs (CD14, CD68, CD163, and CD206). Kaplan–Meier analysis and Cox proportional hazards regression models were applied to estimate overall survival (OS) and recurrence-free survival (RFS).

**Results:**

In UCB tissues, stabilin-1 was primarily expressed on Mφs, as evident from triple immunofluorescence staining for stabilin-1 and Mφ markers. Stabilin-1^+^ Mφs were often more prominent in stromal regions rather than intratumoral regions in UCB tissues (*P* < 0.0001). After dichotomization at the median cell density for stabilin-1^+^ Mφs, only intratumoral stabilin-1^+^ Mφ density was a predictor of poor OS (*P* < 0.001) and RFS (*P* = 0.026). Moreover, intratumoral stabilin-1^+^ Mφ density was positively associated with tumor stage (*P* < 0.01) and histological grade (*P* < 0.01), and emerged as an independent prognostic factor for OS (HR 2.371; *P* < 0.0001), but not for RFS (HR 1.491; *P* = 0.061).

**Conclusions:**

Our findings indicate that intratumoral stabilin-1^+^ Mφs could potentially be used as a pro-tumoral prognostic marker for UCB patients.

**Electronic supplementary material:**

The online version of this article (10.1007/s00345-019-02853-0) contains supplementary material, which is available to authorized users.

## Introduction

Urothelial carcinoma of the bladder (UCB) is the most common type of urological tumor in China [1]. UCB is commonly categorized as non-muscle-invasive bladder cancer (NMIBC) and muscle-invasive bladder cancer (MIBC) [2]. Multiple studies have demonstrated that MIBC has a poorer prognostic outcome than NMIBC [3, 4]. With regard to prognosis, lymph node metastasis and grading systems have been incorporated into prognostic models for predicting the progression of UCB [5]. Recent studies have highlightened the importance of the inflammation, which provides important pro-tumoral function and affects the prognosis of bladder patients [6].

Tumors develop in a very complex microenvironment comprising heterogeneous inflammatory cells for the sustained growth, invasion, and metastasis of tumors [7, 8]. Macrophages (Mφs) are a major component of the leukocyte infiltrate in both mouse and human tumors [9, 10]. Different subsets of tumor-associated Mφs (TAMs) are involved in stimulating angiogenesis/lymphangiogenesis, suppressing antitumor immunity and enhancing tumor cell invasion and metastasis [11]. Diverse specific markers are used to distinguish between Mφ subsets in mice [12], a few of which could be used for the differentiation of human Mφ subsets [12, 13]. In humans, CD14 and CD68 are considered as pan markers of Mφ, but they cannot be used to identify the phenotype or functional status of Mφs [13]. Scavenger receptors of CD206 and CD163 have been used to identify certain pro-tumorigenic TAM populations, and it has been shown that a high density of TAMs is associated with an adverse clinical outcome in the case of most solid cancers [13, 14]. However, a recent study showed that cells with a mature TAM phenotype expressed combinations of previously classified pro-tumorigenic (CD163 and CD206) and anti-tumorigenic (CD169 and CD38) markers in renal cell carcinoma [15]. Therefore, a more detailed understanding of TAM phenotypes in the tumor microenvironment would be useful for investigating and therapeutically targeting these cells.

Stabilin-1 is a type 1 transmembrane receptor and a multi-functional scavenger marker of specialized tissue Mφs; it is involved in receptor-mediated endocytosis, intracellular sorting, and tissue remodeling [16]. Evidence has shown that the tumor microenvironment promotes upregulation of the expression of stabilin-1 in Mφs [17]. Kzhyshkowska et al. demonstrated that stabilin-1^+^ Mφs induced breast tumor growth by mediating silent clearance of extracellular antitumoral factors in mice model [18, 19]. Karikoski et al. found that stabilin-1 could increase immune evasion and lymphatic spread of tumor cells in melanoma and lymphoma [19]. Furthermore, a high density of tumor-infiltrating stabilin-1^+^ Mφs is associated with adverse patient outcomes in breast cancer and colorectal carcinoma [20, 21]. However, little is known about the microlocalization, density, and clinical relevance of stabilin-1^+^ Mφs in human bladder cancer.

Herein, we investigated the distribution and prognostic significance of stabilin-1^+^ Mφs in UCB patients, particularly focused on their anatomic microlocalization. Our data showed that the density of stromal stabilin-1^+^ Mφs is much higher than that of intratumoral stabilin-1^+^ Mφs, but that only intratumoral stabilin-1^+^ Mφ density is a predictor of poor prognosis and is positively associated with tumor stage and histological grade.

## Materials and methods

### Patients and tissue specimens

This study included 283 patients with pathologically confirmed UCB at the Cancer Center of Sun Yat-sen University between Jan 2003 and Dec 2009. All patients underwent cystectomy or transurethral resection for UCB; no patients received any immunotherapy or radiotherapy before surgery, as these pretreatments may affect our results. All the samples were anonymously coded in accordance with the local ethical guidelines (as stipulated by the Declaration of Helsinki). Follow-up information was obtained from the Cancer Center Tumor Registry [22]. Overall survival (OS) was defined as the interval between surgery and death or last observation, and recurrence-free survival (RFS) was defined as the interval between surgery and recurrence or last observation.

### Immunohistochemistry and immunofluorescence analyses

Formalin-fixed and paraffin-embedded samples were processed for immunohistochemical analysis as previously described [23, 24]. After incubation with antibodies against human stabilin-1 (AF3825, R&D system) or control antibody, the sections were incubated with secondary antibodies in an Envision System (Dako).

For triple-color immunofluorescence staining, samples were incubated with sheep anti-human stabilin-1, rabbit anti-human CD14 (10073-R001, Sino Biological) and mouse anti-human CD68 (PG-M1, Dako), or sheep anti-human stabilin-1, rabbit anti-human CD206 (ab64693, Abcam), and mouse anti-human CD163 (ZM-0428, ZSGB-BIO). Images were captured and analyzed on a Zeiss LSM710 system with the ZEN software (Zeiss, Oberkochen, Germany). Single- or double-positive cells of interest in each of two-to-five representative fields at 200 × magnification (0.16 mm^2^ per field) were counted manually by two independent blinded observers. Data are expressed as mean ± SEM to indicate the number of cells per field.

### Immunohistological analysis

The sections were analyzed by two independent observers who were blinded to the clinical outcome. The infiltrating cells in the tissues were categorized according to different regions: intratumoral (INT) and stromal (ST) regions. To evaluate the density of tissue-infiltrating stabilin-1^+^ Mφs, tissue sections were screened under a low-power field (100 ×), and the five most representative fields were selected for analysis at 400 × magnification (0.07 mm^2^ per field). The number of infiltrating cells per field was expressed as the mean ± SEM. The average counts reported by the two investigators were used in subsequent analyses to minimize inter-observer variability.

### Statistical analyses

Statistical analyses were performed using Stata 13.0 (Stata Corp, College Station, TX, USA). The statistical significance of differences between groups was determined using the Wilcoxon signed-rank test. Cumulative survival was calculated using the Kaplan–Meier method and was analyzed by the log-rank test. A multivariate Cox proportional hazards model was used to estimate the adjusted hazard ratios and 95% confidence intervals (CIs), and to identify independent prognostic factors. For categorical analyses, the median value was used as a cutoff to dichotomize continuous variables (for clinical applications). Associations between variables were analyzed using Spearman ρ coefficient tests, and relationships between categorical variables were analyzed using *χ*^2^ tests. For such comparisons, two-tailed *P* values that were < 0.05 were considered to indicate statistical significance.

## Results

### Subpopulation of TAMs in human UCB tissues based on stabilin-1 expression

**Fig. 1:**
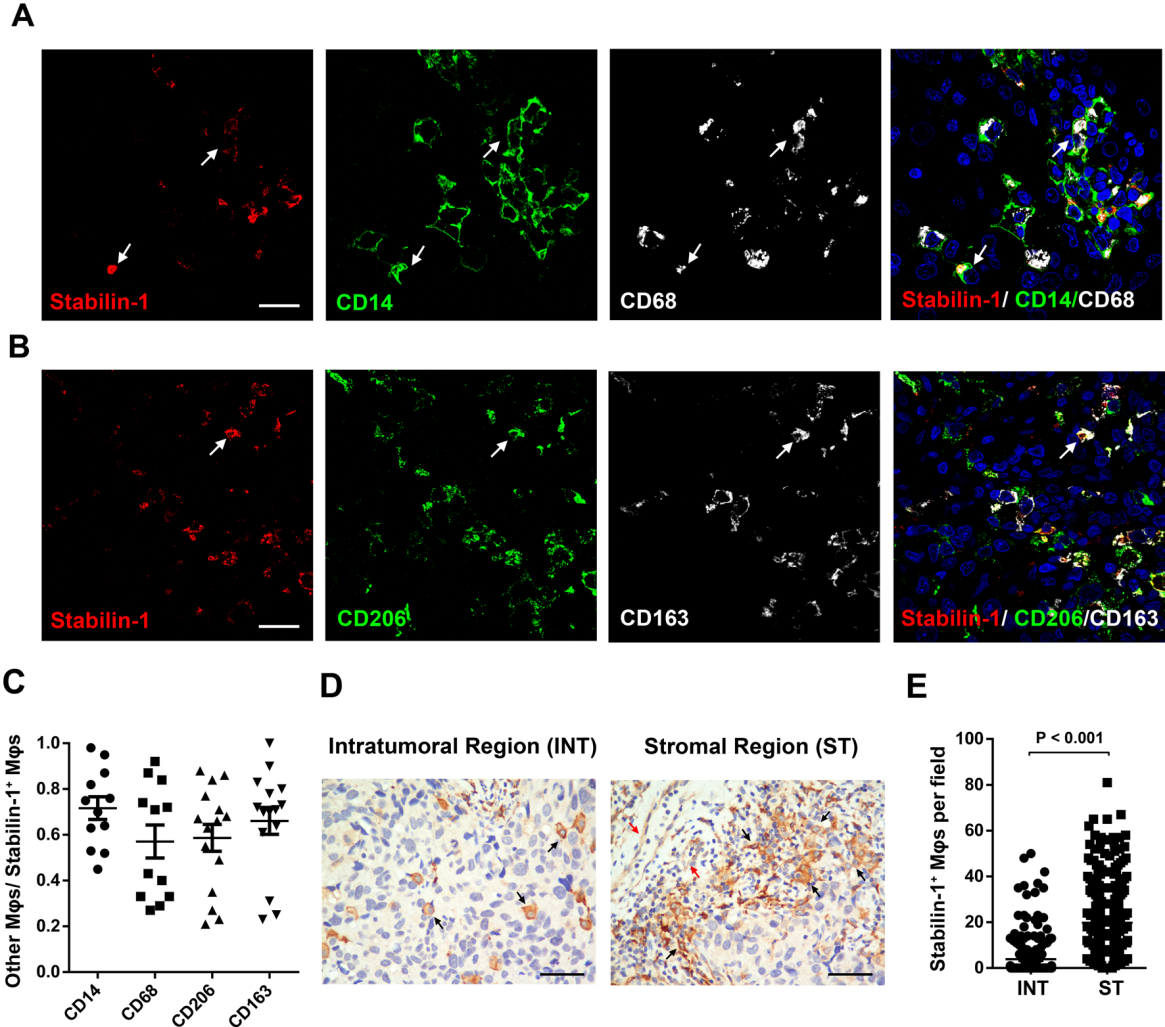
Expression of stabilin-1 and its distribution pattern in human UCB tissues. Paraffin-embedded UCB sections (*n* ≥ 10) were subjected to triple-color immunofluorescence staining for stabilin-1 (red) and either **a** the pan-Mφ marker CD14 (green) and CD68 (white) or **b** the alternative Mφ marker CD206 (green) and CD163 (white), with DAPI counterstaining (blue). The high-power fields show the colocalization of stabilin-1 with the indicated markers (white arrows). Scale bar, 20 µm. **c** Proportion of other Mφs/stabilin-1^+^ Mφs in UCB sections (*n* > 10). Data are expressed as mean ± SEM (bars). **d**, **e** Stabilin-1^+^ Mφs are enriched in the stromal regions of UCB tissues (*n* = 287). **d** Representative immunohistochemistry images of stabilin-1^+^ Mφs in human UCB tissues (black arrows). Stabilin-1 is also expressed on some endothelium cells (red arrows). The micrographs at higher magnification show stained intratumoral regions (INT) and stromal regions (ST). Scale bar, 100 μm. **e** Number of stabilin-1^+^ Mφs in the INT and ST regions of human UCB tissues. Cell numbers were calculated as the cell count per × 400 field. Data are expressed as mean ± SEM values

We used immunostaining to examine whether TAMs express stabilin-1 and their distribution patterns in human UCB tissues. Stabilin-1 was primarily expressed on Mφs, which was demonstrated by co-expression of stabilin-1 and other markers of Mφs (CD14, CD68, CD163, and CD206) in triple-color immunofluorescence stains (Fig. 1a–c). Stabilin-1 was also expressed on some endothelium cells and/or lymphatic vessels in several UCB tissues, but these cells are easily excluded based on morphology (Supplementary Fig. 1; Fig. 1d). Moreover, we found that stabilin-1^+^ Mφs were distributed throughout the tissues and that they were more frequent in stromal regions than in the corresponding intratumoral regions (20 ± 17 and 4 ± 8 cells/field, respectively; Fig. 1d, e).

### Association between stabilin-1^+^ Mφ density and survival in treatment-naïve UCB patients

**Fig. 2:**
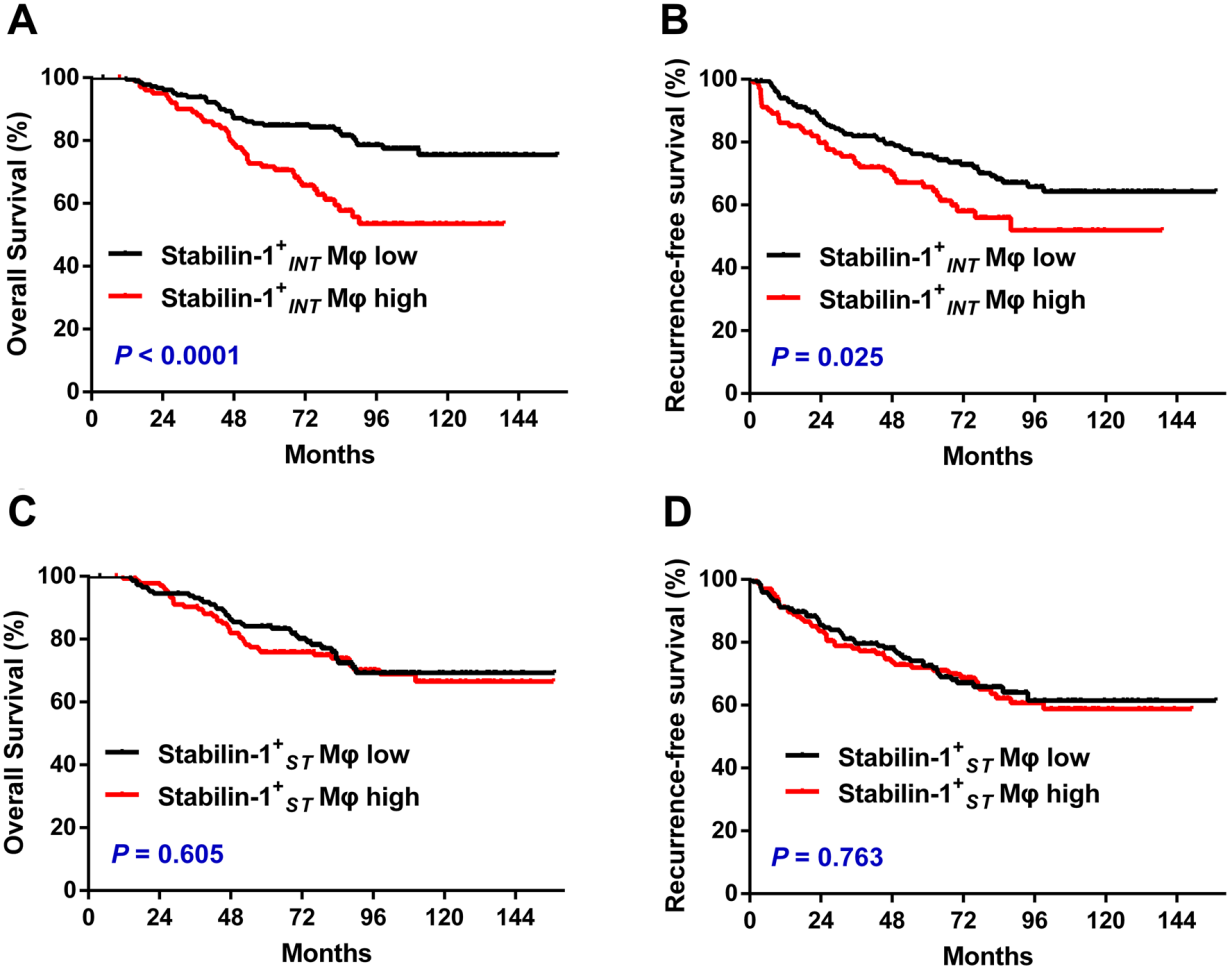
Accumulation of stabilin-1^+^ Mφs in intratumoral regions as a predictor of adverse prognosis in UCB patients (*n* = 283). Patients were divided into two groups according to the median number of cells per × 400 field. **a**, **c** Stabilin-1^+^ Mφs in intratumoral regions (stabilin-$1^{ + }_{\text{INT}}$ Mφs, median = 0). **b**, **d** Stabilin-1^+^ Mφs in stromal regions (stabilin-$1^{ + }_{\text{ST}}$ Mφs, median = 16). The cumulative overall survival time and recurrence-free survival time were calculated using the Kaplan–Meier method and analyzed with the log-rank tests. Black lines, low density of stabilin-1^+^ Mφs; red lines, high density of stabilin-1^+^ Mφs

To investigate the prognostic role of stabilin-1^+^ Mφs in UCB, 283 treatment-naïve UCB patients who had long-term follow-up data (> 10 years) were divided into two groups according to the median counts of stabilin-1^+^ Mφs in the intratumoral regions (stabilin-$1^{ + }_{\text{INT}}$ Mφs) and stromal regions (stabilin-$1^{ + }_{\text{ST}}$ Mφs), respectively. Kaplan–Meier analysis revealed a negative association between the density of stabilin-$1^{ + }_{\text{INT}}$ Mφs and both OS (*P *< 0.001, Fig. 2a) and RFS (*P* = 0.025, Fig. 2b). Patients with a high density of stabilin-$1^{ + }_{\text{INT}}$ Mφs had significantly shorter OS (median, 72 months) and RFS (median, 53 months) than patients with a low density of stabilin-$1^{ + }_{\text{INT}}$ Mφs (median OS, 84 months; median RFS, 72 months). However, the stabilin-$1^{ + }_{\text{ST}}$ Mφ density did not correlate with the survival of UCB patients (Fig. 2c, d). According to the results of univariate analysis, stabilin-$1^{ + }_{\text{INT}}$ Mφ density was associated with both OS and RFS (Table 1). When the clinicopathologic features that were significant in univariate analysis were adopted as covariates (Table 1), multivariate analysis revealed that stabilin-$1^{ + }_{\text{INT}}$ Mφs was an independent prognostic factor for OS (HR 2.371; *P* < 0.0001), but not for RFS (HR 1.491; *P* = 0.061).

**Table 1:** Univariate and multivariate analyses of factors associated with mortality and recurrence in urothelial carcinoma of the bladder (*n* = 283)

Variable	Overall survival				Recurrence			
	Univariate	Multivariate			Univariate	Multivariate		
		HR	95% CI	*P* value		HR	95% CI	*P* value
Age, year (> 60 vs. ≤ 60)	**4.6 × 10**^**−6**^	3.47	2.107–5.713	**< 0.0001**	0.187			NA
Gender (female vs. male)	0.19			NA	0.972			NA
Tumor size (> 3 vs. ≤ 3 cm)	**0.016**	1.286	0.755–2.192	0.354	0.721			NA
Multifocality (multifocal vs. unifocal)	0.947			NA	0.34			NA
Tumor stage (T2–T4 vs. Ta–T1)	**8.5 × 10**^−**7**^	2.292	1.308–4.016	**0.004**	0.964			NA
Nodal status (N1–N2 vs. N0)	**4 × 10**^−**6**^	2.541	1.214–5.315	**0.013**	0.151			NA
Histological grade (G3 vs. G1–G2)	**3.7 × 10**^−**4**^	2.541	0.691–2.026	0.54	**0.007**	1.661	1.099–2.509	**0.016**
Stabilin-$1^{ + }_{\text{INT}}$ Mφ density (high vs. low)	**1.8 × 10**^−**4**^	2.371	1.477–3.803	**< 0.0001**	**0.026**	1.491	0.981–2.265	**0.061**
Stabilin-$1^{ + }_{\text{ST}}$ Mφ density (high vs. low)	0.605				0.763			NA

Univariate and multivariate analyses. Cox proportional hazards regression model. Variables associated with survival by univariate analysis were adopted as covariates in multivariate analyses. Significant *P* values are shown in bold

*UCB* urothelial carcinoma of the bladder, *Stabilin-*$1^{ + }_{INT}$*Mφs* stabilin-1^+^ Mφs in intratumoral regions, *Stabilin-*$1^{ + }_{ST}$*Mφs* stabilin-1^+^ Mφs in stromal regions, *HR* hazard ratio, *CI* confidence interval, *NA* not applicable

HR > 1, higher risk of death; HR < 1, lower risk of death

We also evaluated the influence of stabilin-$1^{ + }_{\text{INT}}$ Mφs in combination with age, tumor stage, and nodal status using the Wald test for interaction (*P*_interaction_). In the multivariate model, combinations of stabilin-$1^{ + }_{\text{INT}}$ Mφ with age (HR 2.095; *P*_interaction_ = 0.001), tumor stage (HR 2.001; *P*_interaction_ = 0.003), and nodal status (HR 1.614; *P*_interaction_ < 0.001) emerged as powerful independent predictors of shorter OS (Table 2).

**Table 2:** Multivariate analysis of combinations of factors associated with UCB patient outcomes (mortality)

Combined variable	Univariate	Multivariate		
	*P*	HR	95% CI	*P*
Stabilin-$1^{ + }_{\text{INT}}$ Mφs and age	**< 0.001**	1.77	1.425–2.199	< 0.001
Low stabilin-$1^{ + }_{\text{INT}}$ Mφ density, age ≤ 60		1		
Low stabilin-$1^{ + }_{\text{INT}}$ Mφ density, age > 60		3.53	1.693–7.355	0.001
High stabilin-$1^{ + }_{\text{INT}}$ Mφ density, age ≤ 60		2.3	1.011–5.239	0.047
High stabilin-$1^{ + }_{\text{INT}}$ Mφ density, age > 60		8.38	3.974–17.651	< 0.001
Stabilin-$1^{ + }_{\text{INT}}$ Mφs and T stage	**< 0.001**	1.7	1.387–2.092	< 0.001
Low stabilin-$1^{ + }_{\text{INT}}$ Mφ density, Ta–T1		1		
Low stabilin-$1^{ + }_{\text{INT}}$ Mφ density, T2–T4		2.22	1.133–4.358	0.02
High stabilin-$1^{ + }_{\text{INT}}$ Mφ density, Ta–T1		1.98	1.030–3.795	0.04
High stabilin-$1^{ + }_{\text{INT}}$ Mφ density, T2–T4		6.23	3.318–11.689	< 0.001
Stabilin-$1^{ + }_{\text{INT}}$ Mφs and nodal status	**< 0.001**	1.61	1.294–2.013	< 0.001
Low stabilin-$1^{ + }_{\text{INT}}$ Mφ density, N0		1		
Low stabilin-$1^{ + }_{\text{INT}}$ Mφ density, N1–N2		4.02	1.493–10.832	0.006
High stabilin-$1^{ + }_{\text{INT}}$ Mφ density, N0		2.56	1.566–4.187	< 0.001
High stabilin-$1^{ + }_{\text{INT}}$ Mφ density, N1–N2		5.28	1.933–14.431	0.001

Univariate and multivariate analyses. Cox proportional hazards regression model. Variables associated with survival by univariate analysis were adopted as covariates in multivariate analyses. Significant *P* values are shown in bold

*UCB* urothelial carcinoma of the bladder, *Stabilin-*$1^{ + }_{INT}$*Mφs* stabilin-1^+^ Mφs in intratumoral regions, *HR* hazard ratio, *CI* confidence interval, *NA* not applicable

HR > 1, higher risk of death; HR < 1, lower risk of death

### Association of stabilin-1^+^ Mφ density in distinct microlocations with tumor progression

Among the 283 treatment-naïve UCB patients, 86 patients (31.4%) had MIBC and 117 patients (41.3%) had high histological grade tumors (Supplementary Table 1). The density of stabilin-$1^{ + }_{\text{INT}}$ Mφs, but not stabilin-$1^{ + }_{\text{ST}}$ Mφs, was positively associated with tumor stage (*P* = 0.005) and histological grade (*P* = 0.005, Supplementary Table 2). Lymph node metastasis (N1–N2) was confirmed in 16 out of the 283 UCB treatment-naïve patients (5.7%). The density of neither stabilin-$1^{ + }_{\text{INT}}$ Mφs nor stabilin-$1^{ + }_{\text{ST}}$ Mφs was significantly associated with lymphatic metastasis.

## Discussion

In the present study, we found that stabilin-1 and other markers for Mφs (CD14, CD68, CD163, and CD206) could be partly co-expressed on the same Mφs, and that stabilin-1^+^ Mφs were distributed more prominently in the ST than in the INT regions in UCB tissues. However, a high density of stabilin-$1^{ + }_{\text{INT}}$ Mφs, but not stabilin-$1^{ + }_{\text{ST}}$ Mφs, was found to be a predictor of poor prognosis in UCB patients, and was positively associated with tumor stage and pathologic grade. These data provide new insights into the significance of the microlocalization, density, and functional orientation of stabilin-1^+^ Mφs in UCB tumor progression.

Human tumor tissues can be anatomically classified into intratumoral and stromal regions, and each has distinct compositional and functional properties [25, 26]. TAMs have various functions according to their microlocalization [25, 27]. Therefore, we focused on the tissue microlocalization of stabilin-1^+^ Mφs in UCB tissues. Our data showed that the density of INT-infiltrating stabilin-1^+^ Mφs emerged as an independent predictor of prognosis. In accordance with our results, the previous studies have also shown that a high density of tumor-infiltrating stabilin-1^+^ Mφs is associated with worse prognosis in colorectal and breast cancers [21, 28]. However, Boström et al. suggested that the stabilin-1^+^ Mφ density is not associated with survival in a group of 184 UBC patients [29]. The contrasting results are probably a result of differences in the tumor stage, number, and size of tumors, as death and recurrence have vastly different probabilities depending on tumor stage. Another important difference is that the Boström study did not evaluate the distribution of Mφs in different microlocations of UCB tissues. However, in our study, only high density of INT-infiltrating stabilin-1^+^ Mφs could predict poor survival in UCB patients. Furthermore, our previous study showed that the density of ST-infiltrating CD204^+^ Mφs, but not INT-infiltrating CD204^+^ Mφs, was a sign of poor prognosis in treatment-naïve UCB patients [23]. Gao et al. have demonstrated PD-L1 and VISTA expression on predominantly distinct subsets of CD68^+^ Mφs representing individual subsets of inhibitory cells in tumor tissues from prostate cancer patients who underwent checkpoint immunotherapy [30]. Their findings indicated that Mφ subsets express distinct markers and show contrasting localization patterns in different tumors.

Stabilin-1 has been established as a good marker for alternatively activated Mφs [20]. However, its functional role in tumor progression is still unclear [31]. Stabilin-1 expressed on Mφs can mediate leukocyte–endothelial contacts and help Mφs and regulatory T cell immigration to tumors [19, 32]. Recent study showed stabilin-1^low^ monocyte population and anti-stabilin-1 Ab-treated monocytes both supported enhanced generation of Th1-dominant immune responses [33]. Moreover, studies on mouse models have shown that the expression of stabilin-1 induced tumor growth and helped tumor cells acquire invasive capability by endocytosis of antitumoral matricellular glycoprotein SPARC in breast tumors [18]. It has also been shown that stabilin-1-deficient mice had smaller primary and metastatic tumors than wild-type controls [19]. Consistent with these observations, our study showed that stabilin-1^+^ Mφs in INT regions were positively associated with tumor stage and histological grade. However, this parameter was not associated with lymph node metastasis; this is probably a result of the low number of lymph node metastasis events and lower statistical power of the findings.

## Conclusion

Our findings indicate that stabilin-1, which is considered a potent pro-tumoral Mφ phenotype marker. The density of stabilin-$1^{ + }_{\text{INT}}$ Mφs was positively correlated with disease progression and stabilin-1 could potentially be used as a prognostic marker for UCB patients.

## Electronic supplementary material

Below is the link to the electronic supplementary material.

Supplementary material 1 (DOCX 1059 kb)
