# Supplementary material for: Microlocalization and clinical significance of stabilin-1+ macrophages in treatment-naïve patients with urothelial carcinoma of the bladder
Source: World J Urol. 2019 Jul 13;38(3):709–16. doi: 10.1007/s00345-019-02853-0 (PMC7064462; doi:10.1007/s00345-019-02853-0)
Supplement: Supplementary file 1 — Supplementary material 1 (DOCX 1059 kb) [file 345_2019_2853_MOESM1_ESM.docx]

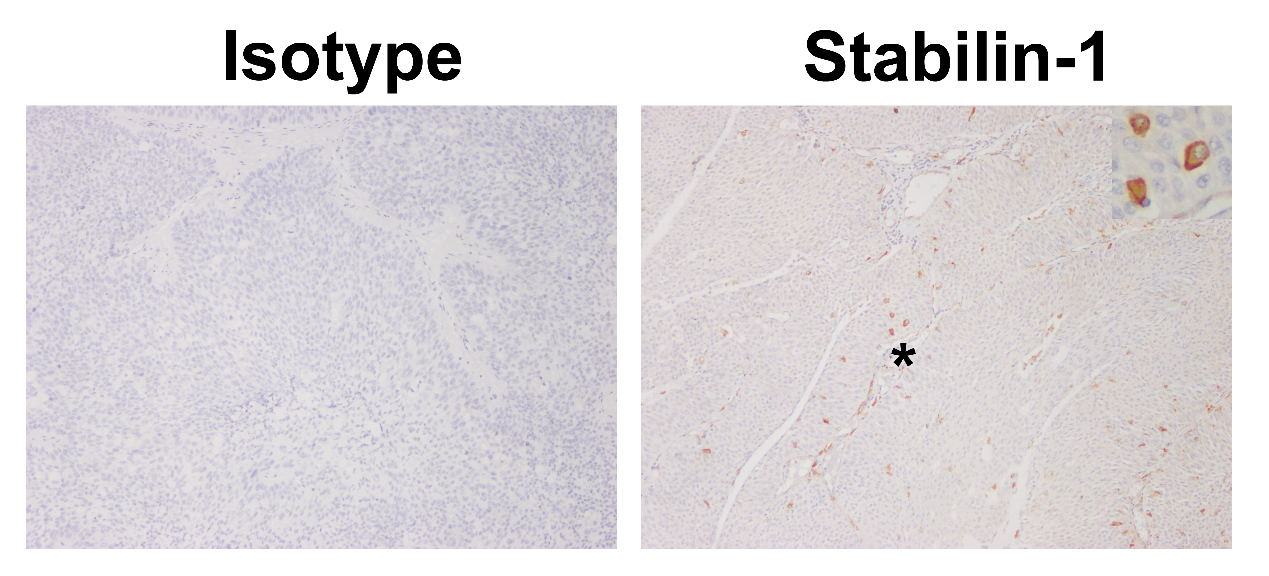


**Supplementary Figure 1. Expression of stabilin-1 in human UCB tissues.** Representative immunohistochemistry images of isotype control and stabilin-1 antibody staining in human UCB tissues. Asterisk indicates the area with higher magnification in the upper-right corner.

**Supplementary Table 1. Clinicopathological Characteristics of Patients with urothelial cell carcinoma of the bladder**

| Variable | No. (%) |
| --- | --- |
| No. of patients | 283 |
| Age, years (median, range) | 60 (15-90) |
| Gender (male/female) | 246/37 (86.9/13.1) |
| Tumor size (≤3 cm / >3 cm) | 219/64 (77.4/22.6) |
| Multifocality (unifocal/multifocal ) | 201/82 (71/29) |
| Tumor stage (Ta–T1/T2–T4) | 197/86 (69.6/30.4) |
| Nodal status (N0/N1–N2) | 267/16 (94.3/5.7) |
| Histological grade (low/high) | 166/117 (58.7/41.3) |
| Follow-up, months (median, range) | 80.3 (4-157) |

**Supplementary Table 2. Associations between Stabilin-1^+^Mφ Density and Clinicopathological Characteristics of UCB**

| **Variable** | **Stabilin-1^+^_INT_ Mφ density** | | | |  | **Stabilin-1^+^_ST_ Mφ density** | | | |
| --- | --- | --- | --- | --- | --- | --- | --- | --- | --- |
|  | **Low** | **High** | **R** | **P** |  | **Low** | **High** | **R** | **P** |
| No. of patients | 182 | 101 |  |  |  | 147 | 136 |  |  |
| Age, years |  |  | -0.008 | 0.894 |  |  |  | 0.08 | 0.18 |
| ≤ 60 | 94 | 53 |  |  |  | 82 | 65 |  |  |
| > 60 | 88 | 48 |  |  |  | 65 | 71 |  |  |
| Gender |  |  | -0.048 | 0.419 |  |  |  |  | 0.668 |
| Male | 156 | 90 |  |  |  | 129 | 117 | 0.026 |  |
| Female | 26 | 11 |  |  |  | 18 | 19 |  |  |
| Tumor size |  |  | -0.05 | 0.401 |  |  |  | 0.089 | 0.137 |
| ≤ 3cm | 138 | 81 |  |  |  | 119 | 100 |  |  |
| > 3cm | 44 | 20 |  |  |  | 28 | 36 |  |  |
| Multifocality |  |  | 0.061 | 0.309 |  |  |  | 0.009 | 0.877 |
| Unifocal | 133 | 68 |  |  |  | 105 | 96 |  |  |
| Multifocal | 49 | 33 |  |  |  | 42 | 40 |  |  |
| Tumor stage |  |  | 0.165 | **0.005** |  |  |  | 0.056 | 0.344 |
| Ta–T1 | 137 | 60 |  |  |  | 106 | 91 |  |  |
| T2–T4 | 45 | 41 |  |  |  | 41 | 45 |  |  |
| Nodal status |  |  | 0.073 | 0.22 |  |  |  | -0.082 | 0.167 |
| N0 | 174 | 93 |  |  |  | 136 | 131 |  |  |
| N1–N2 | 8 | 8 |  |  |  | 11 | 5 |  |  |
| Histological grade |  |  | 0.168 | **0.005** |  |  |  | -0.003 | 0.957 |
| Low | 118 | 48 |  |  |  | 86 | 80 |  |  |
| High | 64 | 53 |  |  |  | 61 | 56 |  |  |

Abbreviations: UCB, urothelial cell carcinoma of the bladder; Stabilin-1^+^_INT_ Mφs, Stabilin-1^+^ Mφs in intratumoral regions; Stabilin-1^+^_ST_ Mφs, Stabilin-1^+^ Mφs in stromal regions.

Significant P-values are shown in bold font.
